# Evaluation of a proteomic signature coupled with the kidney failure risk equation in predicting end stage kidney disease in a chronic kidney disease cohort

**DOI:** 10.1186/s12014-024-09486-5

**Published:** 2024-05-18

**Authors:** Carlos Raúl Ramírez Medina, Ibrahim Ali, Ivona Baricevic-Jones, Moin A. Saleem, Anthony D. Whetton, Philip A. Kalra, Nophar Geifman

**Affiliations:** 1 Stoller Biomarker Discovery Centre, Faculty of Biology, Medicine and Health, The University of Manchester, Manchester, UK; 2 Salford Royal Hospital, Northern Care Alliance Foundation NHS Trust, Salford, UK; 3 Division of Cardiovascular Sciences, The University of Manchester, Manchester, UK; 4 Bristol Renal and Children’s Renal Unit, Bristol Medical School, University of Bristol, Bristol, UK; 5 Veterinary Health Innovation Engine (vHive), Faculty of Health and Medical Sciences, University of Surrey, Guildford, UK; 6 School of Health Sciences, Faculty of Health and Medical Sciences, University of Surrey, Guildford, UK

**Keywords:** End-stage renal disease (ESRD), Chronic kidney disease (CKD), Proteomics, SWATH-MS, Biomarkers, Kidney Failure Risk Equation (KFRE), Actin cytoskeleton pathway, RHO GTPasses, Tight junction

## Abstract

**Background:**

The early identification of patients at high-risk for end-stage renal disease (ESRD) is essential for providing optimal care and implementing targeted prevention strategies. While the Kidney Failure Risk Equation (KFRE) offers a more accurate prediction of ESRD risk compared to static eGFR-based thresholds, it does not provide insights into the patient-specific biological mechanisms that drive ESRD. This study focused on evaluating the effectiveness of KFRE in a UK-based advanced chronic kidney disease (CKD) cohort and investigating whether the integration of a proteomic signature could enhance 5-year ESRD prediction.

**Methods:**

Using the Salford Kidney Study biobank, a UK-based prospective cohort of over 3000 non-dialysis CKD patients, 433 patients met our inclusion criteria: a minimum of four eGFR measurements over a two-year period and a linear eGFR trajectory. Plasma samples were obtained and analysed for novel proteomic signals using SWATH-Mass-Spectrometry. The 4-variable UK-calibrated KFRE was calculated for each patient based on their baseline clinical characteristics. Boruta machine learning algorithm was used for the selection of proteins most contributing to differentiation between patient groups. Logistic regression was employed for estimation of ESRD prediction by (1) proteomic features; (2) KFRE; and (3) proteomic features alongside KFRE.

**Results:**

SWATH maps with 943 quantified proteins were generated and investigated in tandem with available clinical data to identify potential progression biomarkers. We identified a set of proteins (SPTA1, MYL6 and C6) that, when used alongside the 4-variable UK-KFRE, improved the prediction of 5-year risk of ESRD (AUC = 0.75 vs AUC = 0.70). Functional enrichment analysis revealed Rho GTPases and regulation of the actin cytoskeleton pathways to be statistically significant, inferring their role in kidney function and the pathogenesis of renal disease.

**Conclusions:**

Proteins SPTA1, MYL6 and C6, when used alongside the 4-variable UK-KFRE achieve an improved performance when predicting a 5-year risk of ESRD. Specific pathways implicated in the pathogenesis of podocyte dysfunction were also identified, which could serve as potential therapeutic targets. The findings of our study carry implications for comprehending the involvement of the Rho family GTPases in the pathophysiology of kidney disease, advancing our understanding of the proteomic factors influencing susceptibility to renal damage.

**Supplementary Information:**

The online version contains supplementary material available at 10.1186/s12014-024-09486-5.

## Background

Chronic kidney disease (CKD) is an increasing global public health concern. It poses a major challenge to healthcare systems due to its rising incidence and prevalence in various regions [1, 2]. In England, a 2020 assessment revealed that 7.3% of adults are affected by CKD stages 3–5 [1]. The societal impact of renal disease is substantial, manifesting in considerable healthcare costs and imposing burdens on patients and their families. In the National Health Service (NHS) in England, CKD is estimated to account for approximately 2% of the total budget [3]. Even though the population receiving renal replacement therapy (RRT) constitutes only a small fraction—one in fifty of those diagnosed with CKD—, their resource utilization constitutes more than half of the projected total expenditure [3]. Signs and symptoms in end-stage renal disease (ESRD) are often non-specific and might not appear until irreversible kidney damage has already occurred [4]. Accurate prediction of ESRD is fundamental to provide optimal CKD patient care, as it allows for targeted treatment of those patients with a higher risk [5]. Traditionally, static eGFR-based cut-offs were employed as criteria for care delivery decision making, but more recently, the Kidney Failure Risk Equation (KFRE) has been proven to be a superior tool for predicting the 2- and 5-year risk of developing ESRD in patients with CKD stages 3a-5 [6]. Its accuracy has been demonstrated and validated in various international studies [7]. In clinical care systems, absolute risk thresholds based on the KFRE have been adopted to guide treatment decisions. In the UK, the National Institute for Health and Care Excellence (NICE) recommends using the KFRE in primary care to identify patients with a high risk of ESRD who may benefit from early referral to specialist services. The KFRE comes in two versions: a 4-variable model and an expanded 8-variable model. The 4-variable KFRE considers factors such as age, sex, estimated glomerular filtration rate (eGFR) and albuminuria, whilst the 8-variable KFRE includes four additional parameters: serum calcium, phosphate, albumin, and bicarbonate [6]. In a validation study conducted by Ali and Kalra in 2021 [5] the KFREs were proven to have a better clinical utility than relying solely on eGFR when making clinical decisions for patients with advanced CKD.

Previously, our research team proposed to investigate whether proteomic signatures of rapidly progressive CKD could be derived [8]. Our findings supported the complement cascade and coagulation pathway playing a role in the development and progression of renal disease. Glycoprotein Afamin (AFM), CCT4 and C6 emerged as promising biomarkers for tracking CKD progression. These results infer the existence of effective biomarker alternatives to traditional diagnostic methods. To build upon previous research [5], in this study we proceeded to evaluate the KFRE in a UK-based advanced CKD cohort, and explored whether improved predictive accuracy could be obtained by utilising a proteomic signature along with the 4-variable UK-calibrated KFRE in the prediction of ESRD development within 5-years. We demonstrate that this is indeed the case.

## Methods

### Study population and setting

The primary cohort of the Salford Kidney Study (SKS; Co-I: Kalra) biobank comprises 3,600 prospectively followed patients with non-dialysis dependent CKD (NDD-CKD) in the United Kingdom. These individuals have given consent for the sharing of clinical data, as well as for the analysis of plasma and serum biomarkers and genomic studies. This longitudinal, ongoing observational study with full ethical approval, has recruited and followed-up patients since March 2002. Patients are monitored until discharge, death, or withdrawal from the study [9]. Average follow-up is currently 40 months and 17% have progressed to end-stage kidney disease. Patient information, including physical characteristics, medical conditions, and laboratory data, is collected at baseline and annually during routine clinic visits. At each visit samples, including EDTA whole blood, serum and citrate plasma are collected, centrifuged, and stored at  –80 °C in the local Biological Repository for biomarker and genomic research. This study included participants aged 18 years or older at the time they provided their consent, and patients who had an estimated glomerular filtration rate (eGFR) below 60 mL/min/1.73 ${m}^{2}$, but had not yet initiated renal replacement therapy.

### Albuminuria, GFR slope calculation and patient selection

During routine clinic visits, serum creatinine levels were measured using a calibrated Jaffe method, traceable to an isotope dilution mass spectrometry reference measurement procedure. This method allowed estimation of the GFR using the Chronic Kidney Disease Epidemiology Collaboration (CKD-EPI) Equation [10], a prerequisite for the new UK-calibrated KFRE. CKD-EPI eGFR values were used to calculate GFR slopes (ΔGFR) [8]. The ΔGFR for each patient was computed using ordinary least-squares linear regression based on all outpatient eGFR values during the follow-up period of the study. Inclusion criteria included: (1) patients who had a minimum of four eGFR measurements over a two-year period; and (2) patients exhibiting a linear eGFR trajectory. To ensure this, two independent researchers visually reviewed each patient’s eGFR-time slopes, a methodology used in previous research [9]. Patients derived from the two ΔGFR groups: rapid decline (> 3 ml/min/year) and stable (- 0.5 to + 1 ml/min/year) CKD. This study encompasses a diverse group of patients with various renal diseases, including diabetic nephropathy, hypertensive nephropathy, autosomal dominant polycystic kidney disease (ADPKD), glomerulonephritis, as well as individuals with 'other' CKD (multiple less frequent diagnoses) or those with unknown cause of CKD [8]. The urine albumin-to-creatinine ratio (uACR) was calculated from urine protein-to-creatinine ratio for all patients using a validated online conversion tool [11]. This conversion tool has been demonstrated to accurately estimate uACR values and is compatible with the KFRE [5].

### Kidney failure risk equation

In the current study, we utilised the new UK-calibrated KFREs, which differ slightly from those employed by our research team in previous publications [12], but are desirable as they are tailored to the UK population. The UK-calibrated KFRE model incorporates an adjustment factor to the original KFRE based on the difference between the prevalence of kidney failure in the Canadian population (used to develop the original KFRE tool) and the prevalence of ESRD in the UK population [13]. The 4-variable UK-calibrated KFRE was calculated for each of the 433 patients based on their clinical features at baseline.

### Outcome

The main outcome for the study, ESRD, was defined as initiating long-term haemodialysis or peritoneal dialysis, receiving a renal transplant, or initiating follow-up in a conservative care clinic within five years from the baseline date.

### Sequential Window Acquisition of All Theoretical Fragment Ion Spectra (SWATH) analysis

Plasma samples were processed and then analysed using SWATH-MS according to our previously published methods [8]. The SWATH-MS analysis was performed using defined mass spectrometry parameters, including isolation window size, overlap and total cycle time, enabled protein-relative quantification of more than 900 proteins, as previously reported [8, 14]. The resulting SWATH map was investigated with reference to clinical data to identify potential blood-borne biomarkers of renal disease. Detailed description of methodologies used for sample preparation, assessment of batch effects, quality control parameters, and SWATH-MS proteomic profiling can be found in the Additional file 1.

### Statistical and data analysis

The proteomic data underwent log2 transformation to stabilise the variance and reduce heteroscedasticity. Negative values arising from the transformation of values smaller than one were considered as missing data. Additionally, proteomic signals that were identified as outliers and exceeded a threshold of 30 were also handled as missing values during the analysis. Any missing values within the proteomic dataset were subsequently replaced with zeros. Downstream analysis using machine learning approaches were performed using the computing environment R (version 4.2.2). Feature selection was performed using Boruta (Boruta version 8.0.0), a wrapper technique built around the random forest classifier. This method compares the importance of the real predictor variables with those of permuted copies of the original features through statistical testing and multiple iterations of random forests. Boruta Feature selection has been applied to SWATH-MS data in various studies [15, 16] and has been shown to be effective and a most stable methodology in permutation-based feature selection [17]. To rank feature importance, the Boruta algorithm employs mean Z-scores, indicating the number of standard deviations a data point deviates from the mean. The higher the Boruta importance score, the stronger the impact of the input variable on the outcome variable [18].

The caret package (version 6.0.93) was employed to create an index with 70% of data to create a balanced training and testing set and stratify the partition by the ESRD outcome. Subsequently, a logistic regression model was constructed to evaluate the performance of the UK-calibrated KFRE in predicting 5-year ESRD. Additionally, we evaluated the performance of the log2-transformed proteomic signature that had been identified as statistically significant through Boruta analysis. The logistic regression model was trained and evaluated with tenfold cross-validation with the aim of measuring its predictive performance using the Receiver Operating Characteristic Area Under the Curve (ROC-AUC) metric. The use of cross-validation helps ensure that the model’s performance is robust and not overly sensitive to the specific data split, making it a reliable approach for assessing its predictive capabilities. The cumulative AUC for the addition of each potential biomarker, in order of its Boruta importance, was calculated using the *Cstat* function from the *DescTools* package (version 0.99.48) and used to evaluate the model performance. ClueGo (version 2.5.7), a plug-in feature in Cytoscape (version 3.8.2) was used to perform enrichment testing using the list of potential biomarkers identified by the Boruta algorithm. In the ClueGo software the following databases were used: GO Biological Process, GO Immune System Process, GO Molecular Functions, REACTOME Pathways, and Wiki Pathways. Only pathways or functions that exhibited an adjusted p-value of < 0.05, calculated using a two-sided hypergeometric test with Bonferroni step-down correction, were taken into consideration. Additionally, a minimum of two proteins/pathway were required for inclusion. For the GO Tree Interval, a minimum level of 4 was set, and a minimum of 2 genes per GO Term/Pathway selection was established. In addition to the above functional analysis, the biomarkers identified by the random forest (RF) algorithm were subjected to functional annotation using the Database for Annotation, Visualisation, and Integrated Discovery (DAVID) tool, with the default Human gene list serving as the background. The biological and molecular significance of each predictor was statistically assessed and adjusted for multiple-testing correction using the Benjamini–Hochberg procedure.

## Results

### Demographic information

The study population consisted of 433 patients from the SKS cohort with a broad range of kidney disease aetiologies, including diabetic nephropathy (n = 88, 20.3%), glomerulonephritis (n = 66, 15.2%), autosomal dominant polycystic kidney disease (n = 49, 11.3%), hypertensive nephropathy (n = 44, 10.2%), other (including several miscellaneous conditions; n = 125, 28.9%) and unknown cause of CKD (n = 54, 12.5%) (Table 1).

**Table 1:** Clinical profile of patients enrolled in the study updated for the 433 patients

Characteristic	Total		Cases (ESRD group)		Controls (No ESRD group)		p-value	
Sample size	n	%	n	%	n	%		
	433	*100%*	141	*100%*	292	*100%*		
Age (years ± SD)	61.5 ± 15.0		55.2 ± 14.5		64.6 ± 14.4		T-test	< 0.001
Gender (n, %)	**n**	***%***	**n**	***%***	**n**	***%***		
Male	270	*62.4%*	86	*61.0%*	184	*63.0%*	Chi-squared test	0.76
Female	163	*37.6%*	55	*39.0%*	108	*37.0%*		
Ethnicity	n	%	n	%	n	%		
White	416	*96.1%*	133	*94.3%*	283	*96.9%*	Chi-squared test	0.30
Other ethnicity	17	*3.9%*	8	*5.7%*	9	*3.1%*		
Diabetes	n	%	n	%	n	%		
	136	*31.4%*	40	*28.4%*	96	*32.9%*	Chi-squared test	0.40
Hypertension	n	%	n	%	n	%		
	408	*94.2%*	136	*96.5%*	272	*93.2%*	Chi-squared test	0.19
Smoking	n	%	n	%	n	%		
	274	*63.3%*	92	*65.2%*	182	*62.3%*	Chi-squared test	0.63
ACE/ARB use	n	%	n	%	n	%		
	306	*70.7%*	99	*70.2%*	207	*70.9%*	Chi-squared test	0.97
Statin use	n	%	n	%	n	%		
	280	*64.7%*	89	*63.1%*	191	*65.4%*	Chi-squared test	0.72
Died	n	%	n	%	n	%		
	134	*30.9%*	43	*30.5%*	91	*31.2%*	Chi-squared test	0.97
ΔGFR (ml/min/1.73 ${m}^{2}$/year)	− 2.1 ± 3.1		− 4.6 ± 3.2		− *1.0* ± *2.3*		T-test	< 0.001
Bicarbonate (mmol/L)	22.5 ± 3.0		21.4 ± 2.6		23.0 ± 3.1		T-test	< 0.001
Creatinine (µmol/L)	204.6 ± 71.7		236.0 ± 83.1		189.3 ± 60.0		T-test	< 0.001
Calcium (mmol/L)	2.3 ± 0.2		2.27 ± 0.2		2.29 ± 0.1		T-test	0.06
Phosphate (mmol/L)	1.1 ± 0.2		1.2 ± 0.2		1.1 ± 0.2		T-test	< 0.001
Albumin (g/L)	42.9 ± 3.7		41.8 ± 4.0		43.5 ± 3.5		T-test	< 0.001
eGFR(ml/min/1.73m^2^)	30.2 ± 14.0		27.0 ± 11.9		31.8 ± 14.7		T-test	< 0.001
uPCR (mg/mmol)	98.0 ± 174.5		189.5 ± 236.1		53.9 ± 111.2		T-test	< 0.001
uACR (mg/mmol)	33.52 ± 81.05		68.9 ± 117.5		16.4 ± 47.0		T-test	< 0.001
Haemoglobin (g/L)	125.2 ± 15.4		121.4 ± 14.2		127.0 ± 15.6		T-test	< 0.001
UK KFRE score (4* variable–5 year)*	0.1 ± 0.2		0.2 ± 0.2		0.1 ± 0.1		T-test	< 0.001

Data presented as mean ± standard deviation (SD) for continuous variables and as counts/percentages for categorical variables. P-values calculated using t-tests for continuous variables and chi-squared tests for categorical variables

eGFR: Estimated Glomerular Filtration Rate; uPCR: Urine Protein-to-Creatinine Ratio; uACR: Urine Albumin-to-Creatinine Ratio; UK KFRE: United Kingdom calibrated Kidney Failure Risk Equation; ΔGFR: Change in Glomerular Filtration Rate; ACE/ARB Use: Use of Angiotensin-Converting Enzyme Inhibitors or Angiotensin Receptor Blockers

The patients in the ESRD group were significantly younger, with a mean age of 55.1 years (SD 15.1) compared to 64.6 years in the non-ESRD group. This age difference was significant (p < 0.001). Furthermore, the ESRD group exhibited significantly lower bicarbonate levels, higher creatinine and phosphate levels, and lower serum albumin and eGFR levels, all of which are indicative of renal dysfunction. While differences in age and various clinical parameters were evident, no significant disparities were observed in sex distribution, ethnicity, prevalence of diabetes, hypertension, smoking status, or the use of ACE/ARB or statin medications between the two groups. The mean UK-KFRE scores for 5-year ESRD prediction were also compared. The difference was highly significant with the ESRD group having higher scores.

### A proteomic signature of ESRD

The resulting SWATH maps quantified a total of 943 proteins in a total of 617 samples (baseline and follow up) from 433 unique patients (Additional file 2: Table S1). After only using baseline samples and analysing the missing values in the dataset, 626 proteins were found to be measurable in at least 20% of the 433 baseline samples and were used for our main analysis. An initial differential expression analysis identified a total of 71 proteins (Additional file 3: Table S2) that exhibited significant differences between the rapid progressor and stable CKD groups (adjusted p-values < 0.05). Using the Boruta Feature Selection algorithm, nine proteins were confirmed as important features relevant for classification based on our ESRD outcome and are presented in ranked order of their mean importance results (Table 2).

**Table 2:** Confirmed proteins by Boruta Feature Selection ordered by Mean Importance

UNIPROT ID	Description	Name	meanImp	medianImp	minImp	maxImp	normHits
P02549	Spectrin alpha chain, erythrocytic 1	SPTA1	27.29	27.27	5.10	38.66	0.97
P60660	Myosin light polypeptide 6	MYL6	27.04	26.94	8.08	40.78	0.98
P13671	Complement component C6	C6	26.49	26.36	4.94	39.84	0.98
Q99784	Noelin	OLFM1	25.88	25.96	5.12	36.78	0.97
Q15746	Myosin light chain kinase, smooth muscle	MYLK	24.65	24.72	3.59	37.71	0.97
Q15365	Poly(rC)-binding protein 1	PCBP1	19.68	19.66	6.87	28.84	0.93
Q13045	Protein flightless-1 homolog	FLII	19.43	19.45	4.78	30.33	0.93
O15143	Actin-related protein 2/3 complex subunit 1B	ARPC1B	15.70	15.63	4.71	27.67	0.84
Q92673	Sortilin-related receptor	SORL1	15.38	15.31	4.85	27.86	0.84

The table summarizes the results of Boruta feature selection, a method used to identify important features in a dataset. The columns include: *meanImp* (Mean Importance): Average importance of each feature across Boruta iterations. *medianImp* (Median Importance): Median importance, providing a robust measure of central tendency. *minImp* (Minimum Importance): Minimum observed importance for each feature. *maxImp* (Maximum Importance): Maximum observed importance for each feature. *normHits* (Normalized Hits): Frequency of a feature being deemed important, normalized to the total iterations. Higher values in *meanImp, medianImp, minImp, and maxImp* indicate greater feature importance, while *normHits* reflects the stability of importance across iterations

**Fig. 1:**
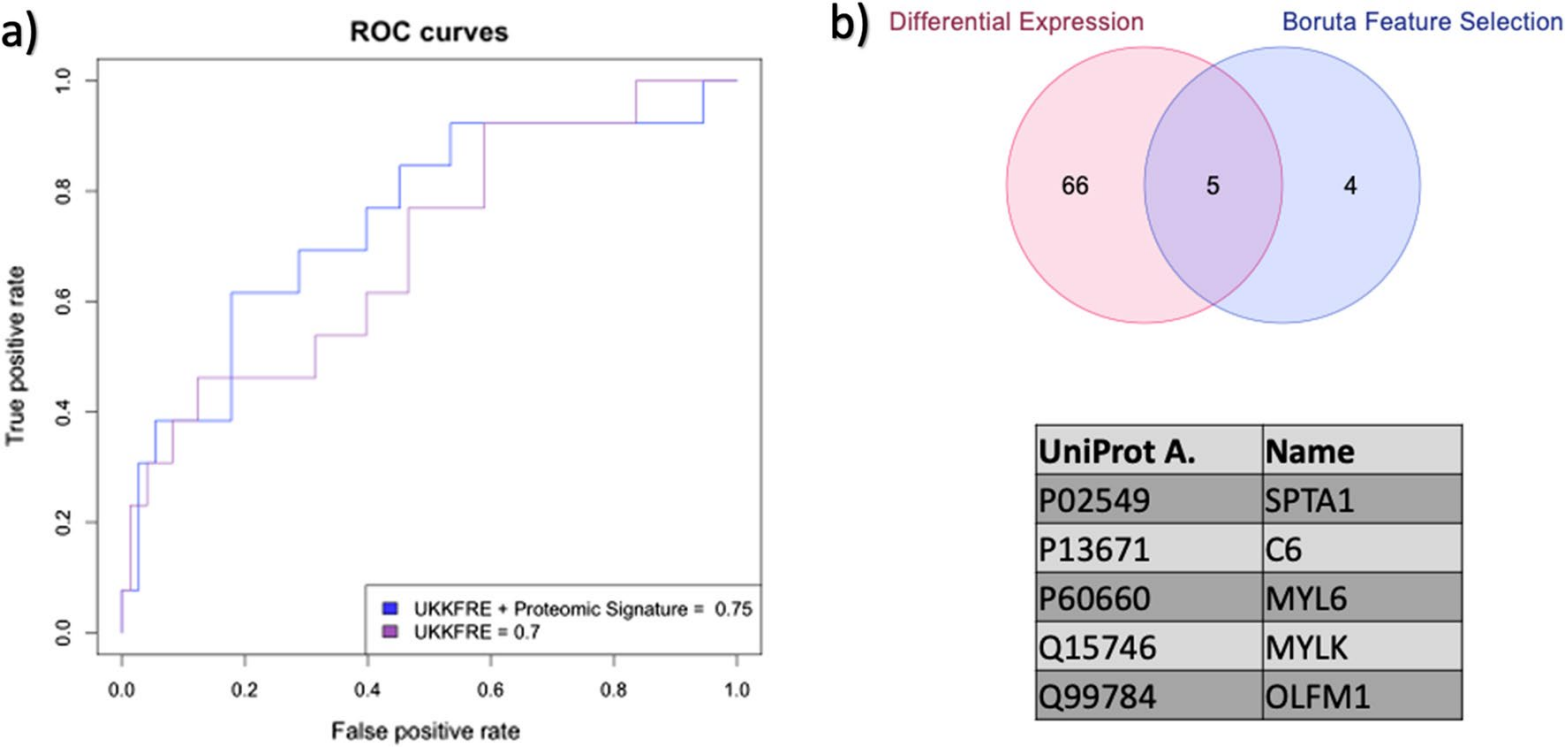
**a** ROC Curves showing the performance of the models built with the top 3 biomarkers identified by Boruta Feature Selection Algorithm. The ROC curve using UKKFRE and proteins SPTA1, C6 and MYL6 gives us the best AUC (0.75).** b** Overlap between the two sets of biomarkers (Significant p-value < 0.05) proteins from differential expression analysis and proteins from Boruta Feature Selection

The logistic regression model constructed utilising solely the UK-calibrated KFRE yielded a performance with a ROC-AUC of 0.70, accuracy of 0.83, aligning closely with findings from previous studies [5, 12]. Expanding the analysis to include the ten confirmed relevant proteins and the 4-variable UK-calibrated KFRE resulted in a marginal improvement in predictive performance compared to the previous model. Specifically, this composite demonstrated an improved AUC of 0.72, accuracy of 0.83. We then developed a simplified model using only the top three proteins identified by Boruta feature selection model: SPTA1, C6 and MYL6. This exhibited an enhanced performance improvement in comparison to the previous models, achieving a ROC-AUC of 0.75 and an accuracy of 0.84 (Fig. 1a).

### Functional enrichment analysis

To identify functional pathways associated with our proteomic signatures, and therefore identify mechanisms that may correlate with progression of CKD, pathway enrichment analysis was carried out using the ten proteins confirmed as important by the Boruta Analysis. Statistically significantly enriched pathways identified by the Database for Annotation, Visualisation, and Integrated Discovery (DAVID) for KEGG pathways and ClueGo functional enrichment (REACTOME pathways) conducted on the Boruta-identified proteins are shown in (Table 3).

**Table 3:** Functionally enriched pathways using ClueGo using the ten confirmed proteins

Category	Term	Genes	Term PValue	Term PValue corrected with Benjamini
REACTOME Pathways	Smooth Muscle Contraction	MYL6 MYLK	< 0.01	< 0.01
REACTOME Pathways	RHO GTPases activate PAKs	MYL6 MYLK	< 0.01	< 0.01
KEGG Pathways	Regulation of actin cytoskeleton	C6 MYLK ARPC1B	< 0.05	0.27
KEGG Pathways	Tight Junction	MYL6 ARPC1B	< 0.05	0.24

MYL6: Myosin light polypeptide 6; MYLK: Myosin light chain kinase; C6: Complement Component 6; ARPC1B: Actin-related protein 2/3 complex subunit 1B

## Discussion

In-depth analysis of the proteome associated with ESRD in a cohort of CKD patients in the United Kingdom has identified a protein signature that leads to modest improvement in predictive performance of ESRD developing within 5-years when combined with the 4 variable UK-calibrated KFRE. With just a subset of three proteins, namely SPTA1, MYL6 and C6, a further slightly enhanced predictive performance is achieved. The value of ROC data has previously been described [19]. The further definition we provide with a three-protein measurement has potential value in patient stratification. ROC can be considered the diagnostic accuracy of a test and thus this increase with an accuracy score of 0.84 (when the reagents required for the biochemical assays are relatively cheap) is a significant step forward. KFRE is employed in clinical decision taking. Kidney Failure Risk Equation (KFRE) has proven to be a superior tool for predicting the 2- and 5-year risk of developing ESRD in patients with CKD stages 3a-5 [6] and has been validated internationally [7]. An enhanced KFRE with three protein assays added then must, by definition, be of clinical value. These results underscore the potential utility of both a set of three protein biomarkers and the 4-variable UK-calibrated KFRE in enhancing the predictive performance of the model for 5-year kidney failure risk assessment.

Enrichment analysis revealed a noteworthy enrichment of biological pathways closely associated to kidney failure. Specifically, these pathways included the “RHO GTPases activate PAKs”, “Regulation of actin cytoskeleton”, and “Tight junctions”. Importantly, among the proteins identified within these enriched pathways were Myosin light chain kinase (MYLK) and Complement component (C6), proteins that previous research has identified as significant biomarkers in CKD progression [8]. This convergence of findings underscores the relevance of these pathways and proteins in the context of CKD and kidney failure.

The analysis of complement component 6 (C6) expression revealed a statistical significant difference between the patient groups (p-value < 0.05, see Additional file 3: Table S2). Patients who developed ESRD displayed lower mean expression levels of C6, suggesting a potential downregulation of this protein as a biomarker of renal disease. C6 plays a critical role in inflammatory responses [20] and serves as a key component of the complement system, whose involvement in the pathogenesis of many kidney diseases is well established [21, 22]. The terminal pathway of complement activation leads to the creation of the membrane attack complex (MAC) which is composed of C5b, C6, C7, C8, and C9 components. The MAC is believed to play an important role in the pathogenesis of diverse kidney diseases by causing cellular injury and tissue inflammation [23]. Of the terminal pathway components, C6 deficiency is the most common component [24]. While complement deficiency is associated with recurrent infections, glomerulonephritis, and inflammatory disorders affecting the kidney and eyes [25], inherited deficiency of C6 has shown to delay the onset of proteinuria and improve renal function in a rat model. Complement may play a dual role in renal disease, exerting both beneficial and harmful effects [22]. This underscores the need for further investigation into the role of C6 in renal disease pathology. In addition, Byglican (BGN), a tissue-derived protein reported to be a biomarker of inflammatory renal diseases [26], was also found to have a statistically significant difference in expression between our patient groups, with elevated levels found in patients that developed ESRD (Additional file 3: Table S2). Expression levels of plasma VCAM1, reported to be associated in urine samples with a variety of inflammatory kidney diseases [27], did not show a statistical significant difference between our patient groups (see Additional file 3: Table S2).

### Regulation of the actin cytoskeleton pathway

Regulation of the actin cytoskeleton is important for the structural integrity of the kidneys.

It is a network of proteins that gives cells their shape and structure and when the actin cytoskeleton is disrupted, it can lead to changes in the shape and function of kidney cells, making the kidneys susceptible to damage. Dysregulation of the actin cytoskeleton in podocytes represents a common pathway in the pathogenesis of proteinuria, spanning a range of CKD conditions [28, 29]. Emerging evidence proposes that interventions aimed at modulating the dynamics of the actin cytoskeleton hold potential in ameliorating podocyte injury and thus, kidney dysfunction [30]. Given the critical role of the actin cytoskeleton in preserving glomerular filtration, understanding the molecular architecture and control mechanisms of actin has become a central focus of investigation in podocyte research [31]. There is evidence that dysregulation of the actin pathway ultimately plays a contributory role in end-stage renal disease (ESRD) [32].

### RHO GTPases pathway

A significant enrichment in the RHO GTPases signalling pathway was also discovered, which is relevant because the RHO GTPases are involved in cell signalling pathways that can lead to kidney inflammation and fibrosis [31, 33]. The Rho family GTPases are molecular switches that play a central role in dynamically regulating the actin cytoskeleton, but also of cellular morphology, motility, adhesion, and proliferation. The activation of the PAKs pathway by Rho GTPases serves as a critical mechanism through which Rho GTPases regulate actin cytoskeleton remodelling and associated cellular processes. Dysregulated activities of the Rho GTPases and of their effectors are implicated in the pathogenesis of both hereditary and idiopathic forms of kidney diseases [34]. Activation of Rho-GTPases has been linked to podocyte dysfunction, the importance of which has already been described in relation to CKD progression [35].

### Tight junction pathway

Tight junctions are important for maintaining the structural and functional integrity of the kidneys, responsible for sealing the cells of the kidney together and playing a vital role in epithelial barrier function [36]. They create a barrier between the cells of the nephron, the functional unit of the kidney, separating and maintaining biological fluid compartments of different composition, and ensuring proper reabsorption and secretion of substances. When tight junctions are disrupted, this can lead to the leakage of fluids and proteins into the renal interstitium, which can cause tubular damage and lead to kidney failure [37].

Certain limitations warrant acknowledgment. Firstly, our inclusion criteria focused solely on patients with linear progression within the rapid progression group. Non-linear decline is common, yet these patients often tend to have different phenotypes and outcomes, and as of yet the proteomic signature of this cohort has not been studied specifically [38]. Additionally, while we carried out internal validation of our findings, and data were analysed using fold-change analysis (which is not affected by overfitting), it is important to acknowledge that machine learning models developed in this study require further validation in independent external samples to demonstrate robustness and generalisability of the results, for clinical utilisation. Such external validation remains pending and should be addressed in future work. Lastly, the analysis of podocyte-specific proteomic data was not within the scope of this study. Incorporating such data into future analyses may contribute to a more comprehensive understanding of the renal disease pathogenesis.

## Conclusions

The proteomic analysis of an advanced chronic kidney disease (CKD) cohort identified that proteins SPTA1, MYL6 and C6, when used alongside the 4-variable UK-KFRE, achieve an improved performance when predicting a 5-year risk of ESRD. Given the international acceptance of the clinical utility of KFRE, our improvement thereon by use of specific protein measurements now requires validation and verification. Specific pathways implicated in the pathogenesis of podocyte dysfunction were also identified, which could serve as potential therapeutic targets.

### Supplementary Information

**Additional file 1:** Supplementary Methods S1.

**Additional file 2: Table S1.** Log 2 Protein identification and quantification data for all individual samples (attached CSV file).

**Additional file 3: Table S2.** Fold Change Differential Expression analysis (attached CSV file).

## Data Availability

Scripts and processed datasets accompanying the paper are available on the github repository: https://github.com/carlosramirezmedina/ESRD_UKKFRE_Proteomics/. The original datasets and additional materials are available from the corresponding author on reasonable request. Further details on data accessibility are available from the Corresponding Author.

## Abbreviations

ADPKD: Autosomal dominant polycystic kidney disease
AFM: Afamin
CKD: Chronic kidney disease
CKD-EPI: Chronic Kidney Disease Epidemiology Collaboration
DAVID: Database for Annotation, Visualisation, and Integrated Discovery
eGFR: Estimated glomerular filtration rate
ESRD: End-stage renal disease
KFRE: Kidney Failure Risk Equation
MS: Mass-Spectrometry
NHS: National Health Service
NICE: National Institute for Health and Care Excellence
NDD-CKD: Non-Dialysis Dependent Chronic Kidney Disease
ROC-AUC: Receiver Operating Characteristic Area Under the Curve
RRT: Renal replacement therapy
SKS: Salford Kidney Study
SWATH: Sequential Window Acquisition of All Theoretical Fragment Ion Spectra
uACR: Urine Albumin-to-Creatinine Ratio
