# Supplementary material for: Evaluation of a proteomic signature coupled with the kidney failure risk equation in predicting end stage kidney disease in a chronic kidney disease cohort
Source: Clin Proteomics. 2024 May 18;21:34. doi: 10.1186/s12014-024-09486-5 (PMC11102163; doi:10.1186/s12014-024-09486-5)
Supplement: Supplementary file 1 — Additional file 1: Supplementary Methods S1. [file 12014_2024_9486_MOESM1_ESM.docx]

Additional files

**Table S1** Log 2 Protein identification and quantification data for all individual samples [attached CSV file].

**Table S2** Fold Change Differential Expression analysis [attached CSV file].

**Methods S1**

**Sequential Window Acquisition of All Theoretical Fragment Ion Spectra (SWATH) Analysis**

Plasma samples were processed and then analysed using SWATH-MS according to our previously published methods (Medina et al, 2023). Briefly, the most abundant plasma proteins were removed by affinity chromatography using Top 12 Abundant Protein Depletion Spin Columns (Pierce Biotechnology, UK) and the eluate was concentrated using Amicon Ultra-0.5 Centrifugal Filter Devices (Merck-Millipore, UK). The depleted plasma was denatured, reduced, and alkylated prior to concentration via vacuum centrifugation. In parallel with individual study samples, a pool of all the study samples, a batch pool for each batch prepared, and a commercially sourced plasma sample (BioIVT, UK) were processed to access data on efficiency in depletion/digestion and for quality control purposes to monitor mass spectrometry performance. In addition, when lyophilised peptide samples were reconstituted, PepCalMix (MS Synthetic Peptide Calibration Kit, AB Sciex UK Ltd, UK) and 10 x index retention time (iRT) standards (Biognosys AG, Switzerland) were added to each sample. iRT peptides contain 11 stable none naturally occurring peptides which are specifically designed to be spiked into LC samples as a form of a quality control for retention times in liquid chromatography prior to the sample reaching the mass-spectrometer and also for longitudinal instrument performance analysis. A batch pool sample and a commercial plasma sample were analysed by polyacrylamide gel electrophoresis (SDS-PAGE) to assay immune-depletion/digestion efficiency.

Samples were analysed by SWATH-MS with a micro-flow LC-MS system comprising an Eksigent nanoLC 400 autosampler and an Eksigent nanoLC 425 pump coupled to a AB Sciex 6600 Triple-TOF mass spectrometer with a DuoSpray Ion Source. Analysis was performed using defined mass spectrometry parameters, including isolation window size, overlap and total cycle time, enabled protein-relative quantification of more than 900 proteins, as previously reported (Medina et al, 2023). Samples were run as duplicate injections with blanks between each sample. Mass spectrometry compatible K562 human protein extract digest sample (Promega, UK), pooled study sample and commercial plasma digest were used to monitor instrument performance. Typically, injection sequences began with the K562 human protein extract digest, followed by commercial plasma digest. For the rest of the injections, after every fourth sample a quality control was acquired, this alternated between the K562 human extract digest and either a digest of a pool of all sample in the study or the commercial plasma digest. Throughout the batch the following parameters were monitored: Total Ion Chromatogram - TIC (to remain 1-2e8 and no drop by more than 20% during the batch; MS1 TIC similar in intensity to total TIC; MS1 TIC and sum off MS2 TICs retained an approximate Gaussian profile).

The first part of the analysis of the SWATH data files was assessment of batch effects. TICs for all quality control samples were compared across all the batches in the study by principal component analysis (PCA). If independent clustering was obtained on samples from the batch or instrument used, then a satisfactory performance was deemed not to have occurred and samples were re-run. The next step was to analyse the coefficient of variance (CV) between the replicates for each sample and to check the total number of proteins quantified. Any sample with a CV% between replicates greater than 20% was also re-run on the mass spectrometer. A minimum of one proteotypic peptide per protein was required for quantification. To ensure data quality, the number of proteins quantified and reported as present must be observed at >30% of samples. The resulting SWATH maps were investigated with reference to clinical data to identify potential blood-borne biomarkers of renal disease as previously seen (Medina et al, 2023).
